# Spontaneous subserosal venous rupture overlying a uterine leiomyoma in a young woman

**DOI:** 10.11604/pamj.2017.28.205.12135

**Published:** 2017-11-07

**Authors:** Amel Achour Jenayah, Sarah Saoudi, Nour Sferi, Rim Skander, Sofiène Ben Marzouk, Abdallah Cherni, Ezzeddine Sfar, Dalenda Chelli, Fethia Boudaya

**Affiliations:** 1 Department “A” of Gynecology and Obstetrics, Center of Maternity and Neonatology of Tunis, Tunisia; 2 Intensive Care Department, Center of Maternity and Neonatology of Tunis, Tunisia

**Keywords:** Hemoperitoneum, leiomyoma, gynaecological emergency

## Abstract

Uterine leiomyomas are very common tumors found in women. Rupture of veins on the surface of uterine leiomyoma is an unusual source of hemoperitoneum. It is an extremely uncommon gynaecological cause of hemoperitoneum. It is a life threatening emergency. We report a case of massive intraperitoneal hemorrhage due to rupture of vessels on the surface of subserous leiomyoma. A differential diagnosis of rupture of leiomyoma’ssurface vessel should be considered, while dealing with a case of hemoperitoneum with pelvic mass.

## Introduction

Uterine leiomyoma is the most common benign tumor in women, with an estimated prevalence of between 20 and 77%. Leiomyomas are usually asymptomatic (50-80%).As a cause of hemoperitoneum, spontaneous rupture of a vein overlying a myoma is extremely rare. Fewer than 100 cases have been reported [1,2]. This is a case report of such.

## Patient and observation

**Figure 1:**
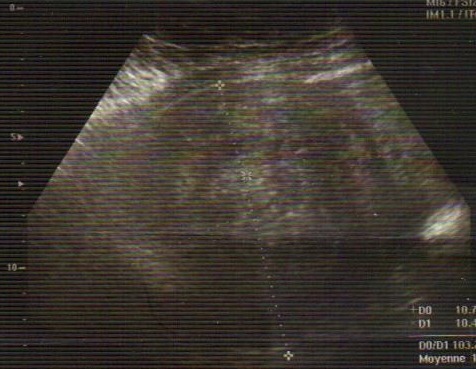
Ultrasound showing 11x10 cm serosal-type fundal uterine myoma

**Figure 2:**
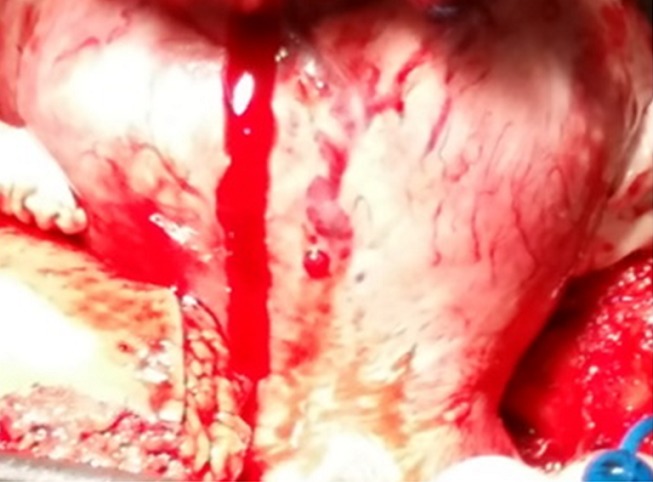
Multiple tortuous veins on the surface of the fundal fibroid

**Figure 3:**
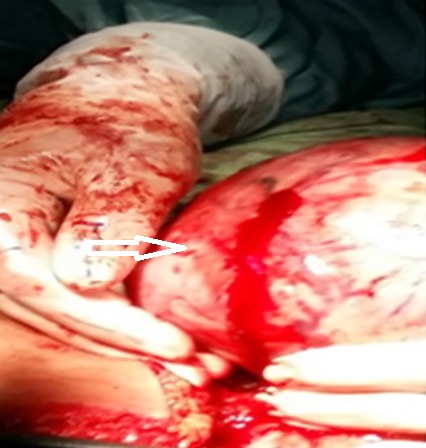
Exploratory laparotomy identified the bleeding site, a ruptured vein located over the dome of the fundal myoma

A 37-years-old woman, G0P0, was admitted to the emergency room complainingof lower abdominal-pelvic pain and generally feeling unwell. No trauma or other gastrointestinal or genitourinary system problems were noted. She had started menses the day of admission. The symptomatology was of rough installation for 10 hours. Clinical examination had foundstable hemodynamicstate, an enormous median abdominal mass arriving up to the navel (consistent with a 20-week gestational uterus) and umbilical and right iliac fossa sensitivity.A pelvic ultrasound was performed. She showed polymyomatous uterus with a 11x10 centimeters serosal-type fundal uterine myoma (Figure 1) and 3 centimeters anterior is thmusmyoma with a small quantity of effusion in the Douglaspouch. Ovaries were not identified. The patient was followed for this polymyomatous uterus and myomectomy was programmed one month later in another hospital.Laboratory data were within normal ranges, hemoglobin level of 11.3 g/dL, blood ßHCGvalue was negative.The first evoked diagnosis was aruptured hemorrhagic ovarian cystbecause of the fast increase of the quantity of the effusion (in 12 hours)found on ultrasound. The decision was an urgent surgical exploration by laparotomy because the big size of the fundal myoma could block the access to the ovaries. To our surprise, ovaries had a normal aspect. Operative findings revealed a 1,000-mL hemoperitoneum. A bleedingsite was identified on the surface of the fundal myoma (Figure 2, Figure 3). This bleeder was derived from a superficial, tortuous and dilated vein.She underwent a myomectomy with a good evolution.

## Discussion

Fibroid of the uterus is the commonest benign tumor seen in women of reproductive age. Spontaneous hemoperitoneum due to rupture of a vein overlying a myoma, however, is a rare entity with less than 100 cases reported in the literature [1]. The first case was reported by Von Rokitansky in 1861 as an autopsy finding. The patient died from peritoneal bleeding [2].The causes given to explain this complication are venous congestion during menstruation, associated with the uterine contractions that can distend the blood vessels, venous congestion during pregnancy, straining during defaecation, any situations leading to increased abdominal pressure (sport, strenuous exercise, massage), abdominal trauma and violent coitus [1,3,4]. In this case; our patient was aware about the presence of fibroid in her uterus. On detailed probing about her symptoms, she narrated that she used to have some pain in abdomen intermittently, which used to subside after rest. In addition, she had her first day of menses on the day she developed symptoms. With regard to the present case, we speculate that an increased congestion of a superficial vein of the fibroid owing to menstruation may have played a part in the vein rupture during the first day of the patient’s cycle. Spontaneous bleeding without any history of trauma, increased abdominal pressure, recent pregnancy or menstruation is extremely rare [2, 4]. The rapid growth of far larger leiomyomas was discussed [1].Ultrasound, although insensitive for small hemoperitoneum and nonspecific in diagnosing the source, is a useful modality and can be performed at the bedside of an unstable patient [5]. Management is both surgical and supportive. Intravenous fluids and blood should be infused for significant blood loss [5].

Treatment aims to stop the haemorrhage and ideallypreserve the uterus in women in childbearing age. When it is possible to make the diagnosis preoperatively,emergency uterine artery embolisationis suggested so that surgery can be scheduledlater.In our case,the patient underwent diagnostic by laparotomy seen the size of the fundal fibroid and the precariousness of her state. She got a myomectomy. She doesn’t receive any units of fresh blood transfusion and had smooth postoperative period. Management of similar cases includes stabilizing the vitalsigns and urgent diagnostic laparoscopy. The laparoscopicapproach is highly recommended in cases of acute abdomen with an uncertain diagnosis.

## Conclusion

Uterine leiomyoma is a frequently seen benign tumor in women of reproductive age group. Bleeding from the rupture of a vein overlying a myomais a rare cause of hemoperitoneumthat we have to keep on mind for patient who consultsfor pelvic pain and known having a fibroid. It may be life-threatening, and needs prompt surgical intervention to stabilize the patient and to establish the diagnosis.

## Competing interests

The authors declare no competing interests.
